# Supplementary material for: Posterior Fossa Approaches Using the Leksell Vantage Frame with a Virtual Planning Approach in a Series of 10 Patients—Feasibility, Accuracy, and Pitfalls
Source: Brain Sci. 2022 Nov 24;12(12):1608. doi: 10.3390/brainsci12121608 (PMC9775269; doi:10.3390/brainsci12121608)
Supplement: Supplementary file 1 [file brainsci-12-01608-s001.zip › PF_Supplements.pdf]

Supplements

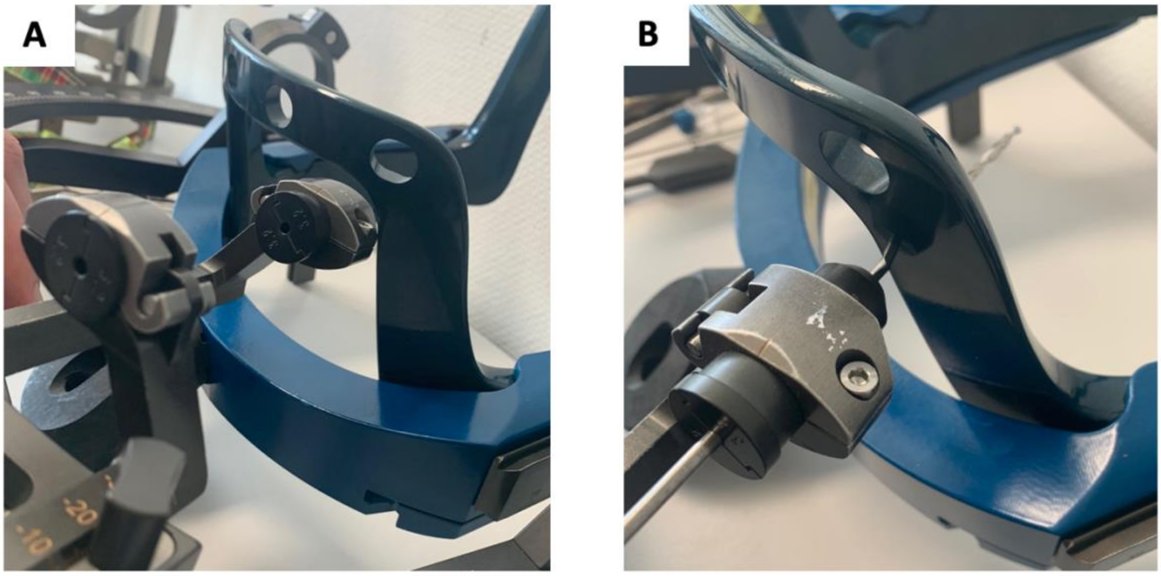

Supplement 1: Feasible ring and arc coordinates if A: complete guide holder is past the frame and B: only the drill bit is past the frame.

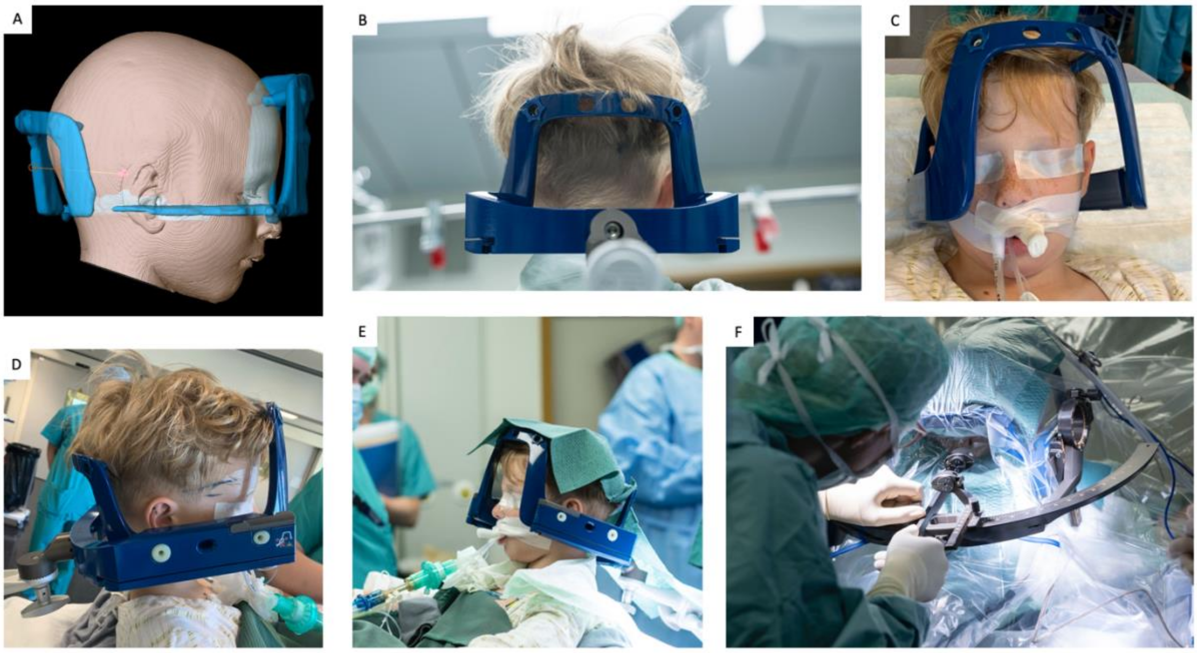

Supplement 2: Example of workflow from planning to intraoperative procedure (patient 3). A: side view of the pre-planned frame placement, B: posterior view, C: anterior view and D: side view of placed frame. E: patient is positioned in a half-sitting position. F: intraoperative view.

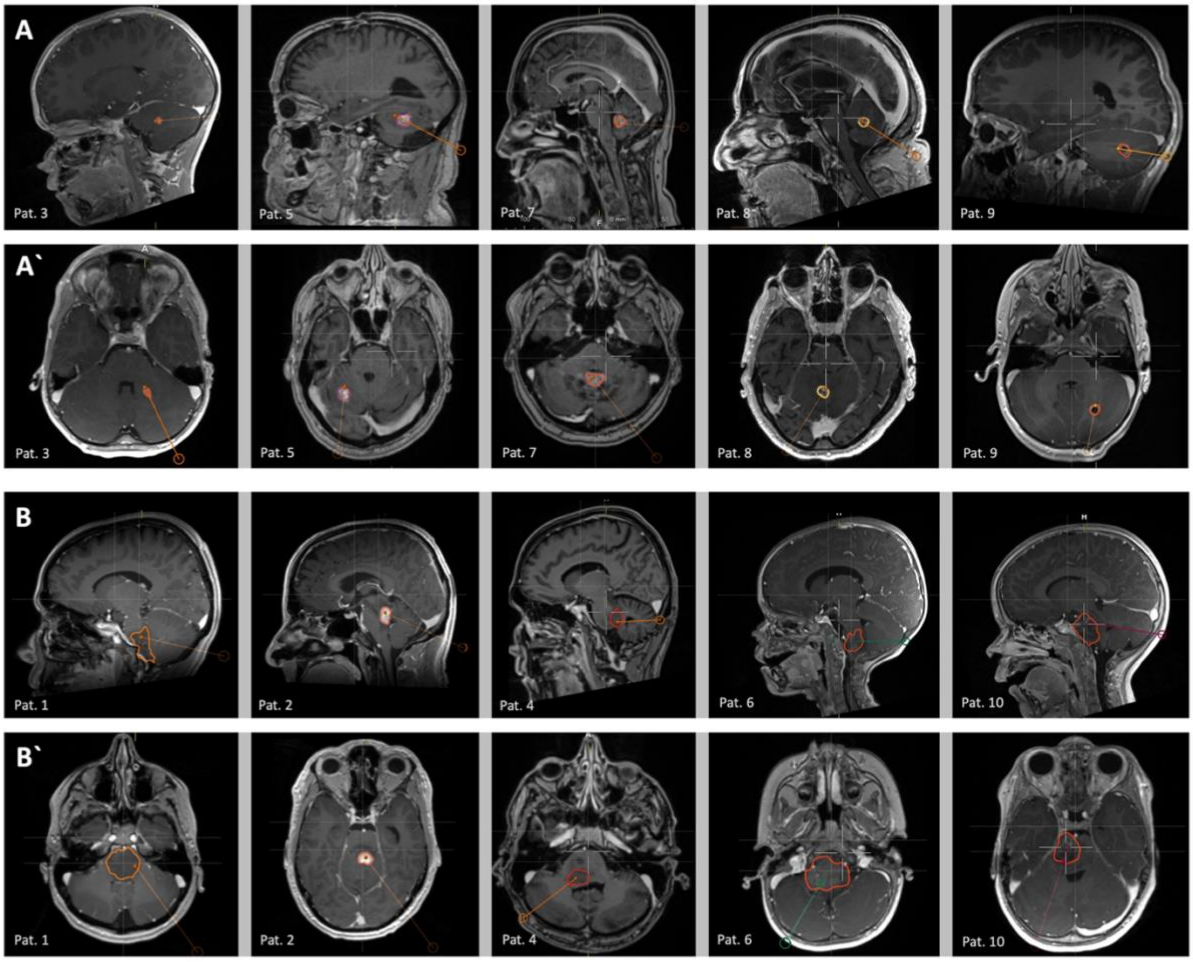

Supplement 3: A: Sagittal view and A`: axial view of cerebellar lesions. B: Sagittal and B`: axial view of brain stem lesions with highlighted lesions and planned trajectories.

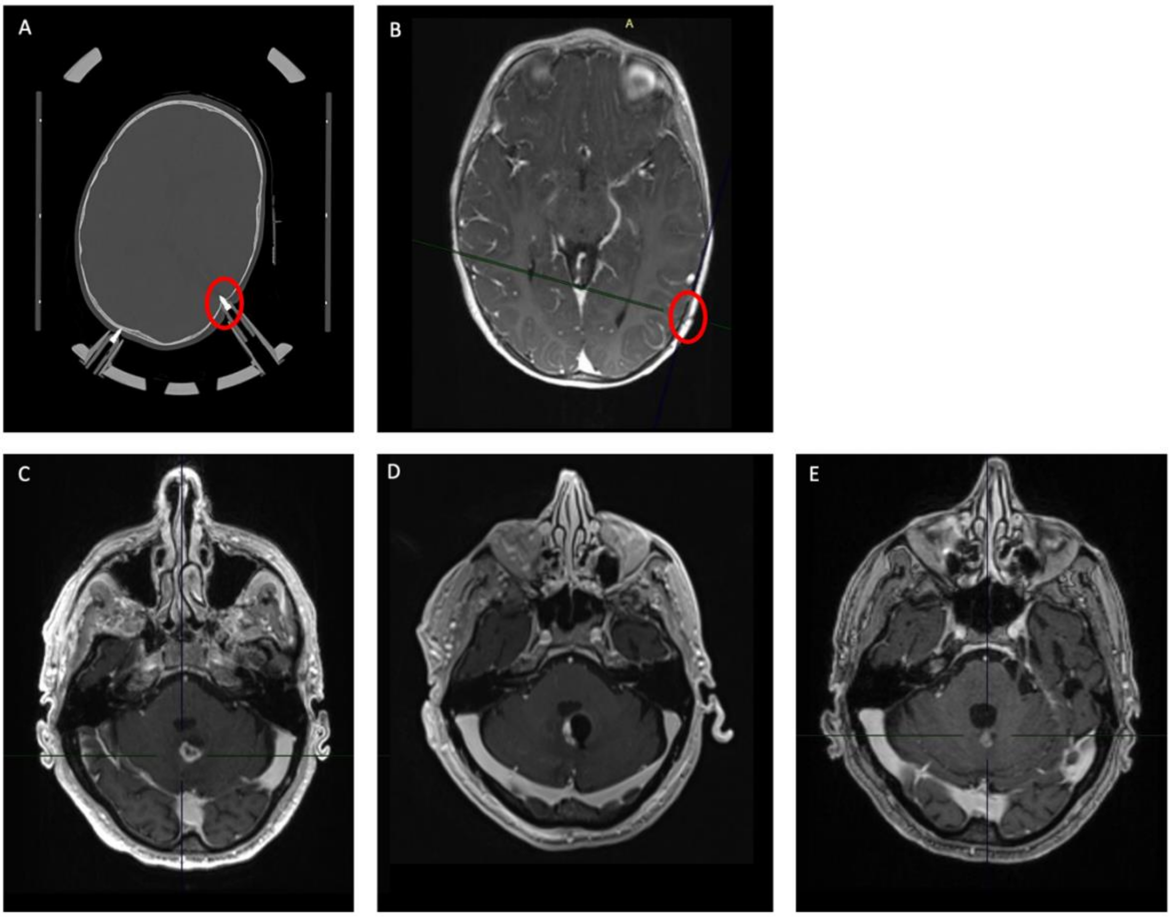

Supplement 4: A: CT showing a fracture caused by pin in two-year old patient. B: postoperative MRI showed no complications. C: preoperative lesion suspected to be a urothelial metastasis. D: Postinterventional haemorrhage seen on MRI scan before initiation of laser ablation procedure. E: 3 months post operative MRI scan shows complete resolution of hematoma and regredient lesion.
